# Relational autonomy in end-of-life care ethics: a contextualized approach to real-life complexities

**DOI:** 10.1186/s12910-020-00495-1

**Published:** 2020-06-30

**Authors:** Carlos Gómez-Vírseda, Yves de Maeseneer, Chris Gastmans

**Affiliations:** 1 Centre for Biomedical Ethics and Law, KU Leuven, Kapucijnenvoer 35/3, 3000 Leuven, Belgium; 2 Faculty of Theology and Religious Studies (Theological and Comparative Ethics), KU Leuven, Sint-Michielsstraat 4 - box 3101, B-3000 Leuven, Belgium; 3 Centre for Biomedical Ethics and Law, KU Leuven, Kapucijnenvoer 35 blok d - box 7001, 3000 Leuven, Belgium

**Keywords:** Euthanasia, Relational autonomy, Decision making, End-of-life, Medical ethics, Palliative Care, Patient Preference, Advance Care Planning

## Abstract

**Background:**

Respect for autonomy is a paramount principle in end-of-life ethics. Nevertheless, empirical studies show that decision-making, exclusively focused on the individual exercise of autonomy fails to align well with patients’ preferences at the end of life. The need for a more contextualized approach that meets real-life complexities experienced in end-of-life practices has been repeatedly advocated. In this regard, the notion of ‘relational autonomy’ may be a suitable alternative approach. Relational autonomy has even been advanced as a foundational notion of palliative care, shared decision-making, and advance-care planning. However, relational autonomy in end-of-life care is far from being clearly conceptualized or practically operationalized.

**Main body:**

Here, we develop a relational account of autonomy in end-of-life care, one based on a dialogue between lived reality and conceptual thinking. We first show that the complexities of autonomy as experienced by patients and caregivers in end-of-life practices are inadequately acknowledged. Second, we critically reflect on how engaging a notion of relational autonomy can be an adequate answer to addressing these complexities. Our proposal brings into dialogue different ethical perspectives and incorporates multidimensional, socially embedded, scalar, and temporal aspects of relational theories of autonomy. We start our reflection with a case in end-of-life care, which we use as an illustration throughout our analysis.

**Conclusion:**

This article develops a relational account of autonomy, which responds to major shortcomings uncovered in the mainstream interpretation of this principle and which can be applied to end-of-life care practices.

## Background

Respect for autonomy is a key principle in contemporary medical ethics [1–3]. The most influential account of this principle derives from *Principles of Biomedical Ethics* [4], a book that has radically transformed medical ethics [5, 6]. The applied-ethics approach of *principlism* lends itself well to translation into ethicolegal standards, such as informed consent. Implementation of informed consent as a tool to express patients’ autonomy is considered indeed to be a major outcome of contemporary bioethics [7, 8].

Regarding end-of-life issues, respect for autonomy is probably the most prominent principle proffered in ethical debates [9, 10]. In discussions about euthanasia, this principle is clearly at the center of the debate, being either praised or criticized [11, 12]. Nevertheless, empirical studies show that a decision-making approach exclusively focused on the individual exercise of autonomy does not align well with patients’ preferences and experiences at the end of life [13, 14]. The need for a more contextualized approach to autonomy, one that meets the real-life complexities experienced in clinical practice, has been repeatedly voiced [9, 15]. In this regard, a relational account of autonomy has been advocated as a more suitable approach [16, 17]. In end-of-life care, relational autonomy has been proposed to be a foundational notion of palliative care [18, 19], shared decision making [20, 21], and advance care planning [22, 23].

However, relational autonomy in end-of-life care is far from being clearly conceptualized or practically operationalized. In 2019, we published a systematic review of argument-based literature that focused on the meaning, foundations, and uses of relational autonomy in end-of-life care ethics; this analysis led to three conclusions [24]. *First*, interpretations of relational autonomy tend to be more of a reaction against an individualistic account of autonomy rather than a positive concept that stands on its own. *Second*, relational autonomy is a rich and complex concept, formulated in complementary ways from different philosophical sources (e.g. feminism, personalism, communitarianism, etc.). However, most of the conceptualizations of relational autonomy refer to a single source of inspiration and present a one-sided interpretation. A richer dialogue between traditionally divergent perspectives could help to clarify the meaning of relational autonomy. *Third*, our review revealed some distance between theoretical approaches of relational autonomy and its operationalization in end-of-life practices. For example, we identified several challenges and limitations whenever relational autonomy had to be translated into practical tools for decision-making in clinical practice.

In this article, we aim to engage in a fruitful dialogue between the lived reality of terminally ill patients and conceptual thinking, vis-à-vis autonomy in end-of-life practices.

## Methods

The starting point of this work is our systematic review on relational autonomy in end-of-life literature [24]. This review showed that relational autonomy is used as a negative concept, one that reacts against the ‘mainstream interpretation’^1^ of the principle of respect for autonomy. A positive development of ‘relational autonomy’ is lacking in the context of end-of-life care.

In order to develop such a positive conceptualization, we opt for an inductive approach, starting from real-life situations. In this regard, we first present an illustrative case, based on clinical reality; we then consider empirical studies that highlight the inadequate response to the complexities of autonomy, as experienced by patients and caregivers in end-of-life care practices. Second, we critically reflect on how relational autonomy can serve as a touchstone for dealing with these complexities, integrating different ethical approaches. In this exercise, we use the case as a validation tool for theoretical ideas to a real situation at the end of life. Finally, we develop a procedural application to the illustrative case. By doing so, we aim at moving forward the dialogue between theory and practice.

## Main body

### Illustrative case^2^

*Mr. Philip is a 45-year-old male patient who was admitted to a palliative care unit in Belgium. He has terminal cirrhosis. In the last 5 years, he has received several chemotherapy regimens targeting an advanced malignant hepatocarcinoma. Because the progression of the disease has advanced unabated in recent months, the patient agreed to be treated palliatively.*

*Upon admission to the palliative care unit, he presents with clear signs of clinical deterioration: edema of the lower limbs, moderate ascites, and mild dyspnea. The patient is conscious, oriented in all three neurological modalities (space, time, and person), lucid, and aware of his poor short-term prognosis. He expresses his willingness to actively participate in decision-making concerning end-of-life care.*

*During his first 3 weeks after admission, he establishes a cordial relationship with the medical and nursing staff. He also speaks frequently with the pastoral care team at the clinic. Mr. Philip is a devoted Christian who has requested spiritual support during his palliative care. His only family is an older sister, with whom he has had a distant relationship during the last years of his life.*

*Mr. Philip’s condition progressively deteriorates. Physical symptoms are tolerable. However, the patient begins to present with several episodes of confusional syndrome, characterized by an acute fluctuating impairment of cognitive function and attention.*

*Being aware of the final stage of his illness, he speaks in-depth to the palliative care doctor and makes a voluntary request for euthanasia. A few days later, he asks to speak with the priest of the service, with whom he has been speaking regularly. Mr. Philip expresses moral uncertainty about his euthanasia decision. After a long discussion with the priest, he changes his mind and puts the euthanasia request on hold. The next day, the patient suffers a self-limited rectal bleeding episode, which visibly affects his emotional state and self-perception. On the same evening, he has an acrimonious argument with his sister during her visit. Mr. Philip calls the priest and the doctor in charge and reinstates his original request: He again decides to go through with the euthanasia.*

### Problematizing autonomy from a reality-based perspective

The case of Mr. Philip reflects the lived reality of decision-making at the end of life in Belgium, a country where euthanasia is legal. The public debate about euthanasia legislation is spreading throughout the world, supported by a certain interpretation of the principle of respect for autonomy. Focusing on clinical reality and not on abstract principles may help to deal more respectfully with the complexity of end-of-life situations. This concrete case presents a patient in a vulnerable situation, characterized by physical and cognitive deterioration. It also brings to fore the relevance of his intimate interactions with different persons who exercise their roles in a highly influential manner. What does it mean to respect Mr. Philip’s autonomous will? Attempts at answering this question make us aware of the complex character of the situation for several reasons. First, it will be difficult to evaluate Mr. Philip’s aptitude for autonomous decisions when his level of rational consciousness fluctuates over time, and his emotional lability affects his capacity for making sound choices. How can one properly assess the patient’s capacity to make autonomous decisions? Second, there is possible risk of external manipulation coming from the priest, the medical doctor, and/or his sister. Who should make the judgement about any possible undue pressure, requested by whom, and based on what criteria? Third, if the healthcare team carries out Mr. Philip’s most recent decision to be euthanized, the patient would no longer have the opportunity to change his mind since euthanasia is an irreversible act. When should the “timer” be stopped and his decision considered to be final? These unanswered questions show that the classical notion of autonomy needs further interpretation and analysis in order to be applied properly in end-of-life situations.

Starting from this case and supported by qualitative research results, we now identify four shortcomings in the ‘mainstream interpretation’ of autonomy that expose the inadequacy of the classical interpretation in fully capturing lived experiences in end-of-life scenarios. Although these four shortcomings are closely related, they can be treated independently of each other and analyzed.

1. ***Autonomy entails more than merely possessing cognitive capacity***

A frequently highlighted shortcoming in clinical practice is to conflate autonomy with decision-making capacity, reducing the latter to a cognitive ability that consists only of understanding information, analyzing it, and communicating a decision based on it to others [25]. An overly zealous emphasis on this sort of capacity has unduly increased the weight that strictly rational^3^ factors have in decision-making processes [22]. To illustrate this problem, Weber et al. found that the exchange of medical information was the main topic of physician discussions on ward rounds, with patients receiving 20 bits of medical information per contact [26]. Bombardment with a high volume of technical information might hinder mutual communication and understanding between patient and doctor.

Yet, even if medical information were adequately dispensed and understood by the patient, it is only one factor among others that impact an eventual decision. Patients at the end of life describe the feeling of being in a “split position,” in which rational arguments and other forces are not always aligned. This scenario is vividly illustrated in the case of a Dutch patient considering euthanasia: “*On the one hand, I definitely want to die. On the other hand, though, there is still simply too much physical, intuitive life force [remaining in me]. (…*) *That’s the dilemma I’m living with: You rationally want to die, but at the same time, there’s that unbreakable will to live, which makes me feel like I’m being pulled in two directions [simultaneously]”* [27]. Another patient in an end-of-life situation summarizes: “*Well, professionally my doctor... I would trust him … forever, and my husband, for what would be best for me … my doctor would use his head, and my husband would use his heart”* [28]. Hence, the presence of rational arguments is considered to be a necessary but insufficient condition for autonomous decision-making to occur.

This first shortcoming highlights the importance of including multidimensional aspects of autonomy when characterizing it; simply considering rational factors leads to an impoverished view of autonomy. Other aspects— such as emotional and embodied factors —are important and deserve specific consideration for inclusion in a real-world understanding of autonomy [29–31].

*In our case, Mr. Philip is a competent and informed person. Most likely, he would perform well on a cognitive test that assesses whether he understands, retains, uses, and weighs the information relevant to a decision and hence would be deemed competent. However, even in the absence of new substantial medical information, his euthanasia decision changes several times, depending on his physical and emotional condition and present state of his relationships with stakeholders. This scenario confirms that an autonomous decision is usually based on more than just cognitive factors that underpin rational thinking.*

2. ***Autonomy is not exercised by patients existing in a social and cultural void***

Mainstream characterizations of autonomy seem to conceive it as playing out in a social and cultural void. However, human beings are not unconnected, solitary atoms; they are embedded in a web of relationships with other human beings and in a concrete cultural context [29]. As related by a palliative care doctor in a qualitative study of terminal cancer patients: “*None of us live in a cocoon … Autonomy is good to a point, but it is not the be all and end all*” [32]. In the same vein, Lister and Campling conclude: “Autonomy, and the agency that derives from it, is only made possible by the human relationships that nourish it and the social infrastructure that supports it” [33].

A too-individualistic interpretation of respect for autonomy, focused solely on protecting the patient against external coercion, has led to the regrettable outcome in which patients are isolated from their social environment, forcing them to decide for themselves [31]. Some authors maintain that, by neglecting the relational dimension of decision-making, an unreasonable burden is placed on patients when making medical decisions [34]. Further, research reveals that the contemporary “autonomy framework” is often seen as a way to protect doctors by transferring the responsibility of decision-making to patients and their families [35].

Studies of oncology patients show that they prefer to share decisions with, or even delegate them to, their doctors [36–38]. As related by a mother when making a decision on behalf of her child: “*I wanted to participate in the decision, knowing that in any case, it is the physician who had the predominant power. Even if they’d asked us our opinion directly, we’d made the decision the way that they were telling us to*” [35]. Similarly, a qualitative study focusing on end-of-life decision-making suggested that, in order to meet patients’ and families’ preferences, physicians should assume more responsibility in recommending treatment plans [28].

In addition, empirical studies show that while some patients wish to have full control in the deliberative process, others prefer to defer decision-making to their family members or, at least, to consider their interests extensively [39, 40]. In end-of-life care, healthcare staff and family are often intimately involved, all being individuals that may affect, and be affected by, the patient’s autonomous decisions [21, 41, 42].

Finally, other studies report that the cultural context strongly influences care relationships and concrete decision-making [15, 43]. For example, ethnicity or common cultural tradition, has proven to be one of the major factors associated with end-of-life preferences [44]. Non-Western patients are not exclusively concerned with making purely autonomous decisions [45, 46]; they are especially attentive to cultural values, such as family harmony, trust, and filial responsibility [24].

This second shortcoming shines a spotlight on the problems associated with a too-individualistic interpretation of autonomy. Such a view fails to capture the clear importance of personal interactions among the patient, relational environment, and healthcare professionals. Finally, all these linkages occur in a particular social and cultural context, and thus need to be considered together in order to arrive at a comprehensive view of autonomy.

*In the case of Mr. Philip, he asks for help and guidance in his decision about euthanasia. He voluntarily consults his medical doctor and the priest. Each one responds from their own perspective, presumably seeking to help Mr. Philip to clarify his preferences and values. Complicating the decision-making process, he changes his mind again after a discussion with his sister. Mr. Philip likely felt pulled in several directions by the connected web of relationships he has with the doctor, the priest, and his sister; yet, he remained unsure of what his own personal preference was concerning his request for euthanasia.*

*In sum, Mr. Philip exercises his autonomy in the context of a diverse and highly influential relational environment. Moreover, these personal interactions take place in the sociocultural context of Belgium, which ascribes certain expectations and constraints to the role played by each stakeholder.*

3. ***Autonomy is not a binary “all-or-nothing” notion***

Mainstream discourse on the autonomy principle tends to characterize it in a binary sense: Either one has it or one does not. If the patient is deemed competent, informed, and free from external pressure, one must strictly follow his/her requests. Yet, if just one of these three conditions is lacking, then an external agent should make decisions on his/her behalf. In this sense, autonomy is considered an “all-or-nothing” notion [47]. However, this characterization does not reflect the real experiences of healthcare professionals working in the field. Very often in end-of-life situations, autonomy is not wholly present or wholly absent but instead is compromised to some degree [25]. In a qualitative study conducted in Australia, a palliative care physician recounts: “*I think it’s a bit overly pretentious to say that a patient has full autonomy when they’re dying. Because, unless others help them, it’s very difficult to be an effective agent for yourself when you’re physically and mentally quite frail*” [32].

When autonomy is represented as a scalar notion along a continuum, patients can be “more or less autonomous”. This scalar property does not belong exclusively to end-of-life frail patients; it is rather an essential property to autonomy as experienced in real life. As Nedelsky points out: “The functioning of the capacity for autonomy is highly fluid: it varies across time and spheres of our lives. Autonomy exists on a continuum. As we act (usually partially) autonomously, we are always in interaction with the relationships (intimate and social-structural) that enable our autonomy” [48]. It is this ‘actual autonomy’, sometimes limited and compromised, which needs to be honored and deserves respect [49]. A relational approach is more sensitive to the support that patients need in order to maintain a certain level of autonomy. Further, a scalar consideration of autonomy does not weaken its normative claim; on the contrary, relational authors demand even more efforts to protect and promote this kind of autonomy “in ethical discourse, in the law, in public policy and professional practice” [50].

To summarize, the current tendency to dichotomize autonomy as either present or absent does not correspond well with actual end-of-life care practices. Autonomy is, therefore, more adequately depicted as a scalar notion.

*In our case, Mr. Philip’s autonomy is partly compromised due to the pain and systemic weakness he is experiencing. Furthermore, at the end stages of his illness, his cognitive state alternates between moments of lucidity and moments of confusion, moving in and out of consciousness, with no clear boundary between these states. From an “all-or-nothing” understanding of autonomy it is difficult to give this compromised autonomy its due place.*

4. ***Autonomy is not exercised in terms of isolated discrete decisions***

Following Beauchamp and Childress’ approach, autonomy tends to be thought of as a characteristic of decisions rather than a characteristic of persons [4]. The option of characterizing medical choices as being static may have practical advantages. For instance, it lends itself well to practical operationalization in implementing the formal standard of informed consent [51]. Nevertheless, autonomy as a lived experience does not get exercised through discrete moments of choice, but rather through a dynamic and interactive process that evolves over time [22].

With such a temporally extended perspective, the relationship between the caregiver and the patient becomes more relevant. As expressed by an oncologist: “… *Usually I’ve known the patients for a long time throughout their illness, and so sometimes, those end-of-life discussions unfold in a very graduated way. And you kind of get to know the patient … over a you know, you develop a relationship and you learn about different aspects of their goals of care, not just the end of life*” [32]. Dealing with a euthanasia request, a Dutch physician explains: “*I think as a doctor, you need to prepare for this [situation] really well, not just at that particular moment [of the request], but you need to start years before. You need to discuss things and document them repeatedly*” [52].

Time is crucial not only from the doctor’s perspective but from the patient’s as well. In an interview study of breast cancer patients, Shih et al. related that, as patients progress with their disease care, they begin to understand medical treatments better, and they gradually develop a sense of self-confidence regarding medical decisions [53]. Shih and colleagues’ study concluded that patients develop heightened awareness of their own preferences and values in care options, and they become more assertive in expressing their current treatment preferences.

This fourth shortcoming of thinking about autonomy as being exercised solely in terms of discrete choices brings into sharp contrast the alternative of treating autonomy as a process when making decisions. Autonomy is better understood as being exercised in terms of a process unfolding over time rather than as a punctate choice fixed in time.

*In our case, a static view of autonomy that focuses on discrete decisions leads us directly to a problematic situation. Depending on when we stop the process and consider a request as the final decision, there are different “autonomous choices,” each one fixed in time. Specifically, Mr. Philip’s decision changes over time, and his choice differs depending at what point in time he has talked with the doctor, the priest, or after suffering a hemorrhage and arguing with his sister. Clearly, a static understanding of autonomy fails to capture the evolving reality of decision-making processes over time.*

These four shortcomings just considered aimed to capture in a synthetic way some of the major criticisms of the mainstream interpretation of individual autonomy. By considering in this analysis the lived experiences that patients and caregivers have at the end of their life, we arrive at a juncture that compels one to look for alternative ways of thinking about autonomy. The alternative approach to a mainstream understanding of the autonomy principle, conceptualized as relational autonomy, is increasingly attracting the attention of ethicists [24].

In the second part of this paper, we present a positive account of relational autonomy, one that incorporates various elements of different ethical approaches to advance the idea that decision-making in end-of-life scenarios is better understood when taking into account social-cultural context and relationships. By doing so, we additionally aim to respond to two major concerns raised by our earlier systematic review on relational autonomy in end-of-life care ethics [24]: first, that relational autonomy is rather a negative, reactionary notion against the individualistic view of autonomy; and second, that despite being a multi-source concept, relational autonomy is currently too often conceptualized from an interpretation that is one-sided.

### A relational account of autonomy

Drawing upon ideas of relational autonomy theories, we will respond to the four shortcomings of an individualistic understanding of autonomy laid bare in the above analysis of Mr. Philip’s case and empirical evidence.

1. ***Autonomy as a multidimensional capacity***

Our first criticism uncovered the problem that the exercise of autonomy cannot be reduced to a unitary rational capacity. End-of-life studies corroborate the scenario depicted in our illustrative case, in which a patient reacts to illness and physical decline with shifting attitudes and behavior over time [54]. This vacillation directly affects their desires and decision-making. From a narrow rationalistic perspective, patients may be perceived as being “inconsistent” in their wishes; sometimes they may be labelled as incompetent, or even as harboring pathological cognition. However, from a broader, more comprehensive perspective that takes into account many dimensions of the patient, what initially might seem like “contradictory” statements actually can be understood as part of the decision-making process unfolding over time. Ambivalence is part of one’s personal life history and moral experience, both rarely fitting into a neat straightforward rational explanation. Respecting complexity at the end of life demands that ethicists and healthcare providers acknowledge non-rational dimensions of a person and integrate these in ways that lead to more authentic choices from the perspective of the patient’s history [55].

In fact, rational capacity is only one of the necessary conditions for an action to be considered autonomous. Beauchamp and Childress already distinguished three conditions for truly autonomous actions, which go beyond the cognitive: intentionality, understanding, and noninterference from external agents [4]. Recent philosophical literature has largely expanded the necessary conditions of autonomy [17, 51]. An exhaustive presentation of this debate is beyond the scope of this paper. However, a cogent presentation is put forward by the major relational autonomy theorist, Catriona Mackenzie. She distinguishes three interdependent dimensions of autonomy, each one including different conditions [56]. This framework offers a taxonomy that can serve as a practical “map” for the field of end-of-life ethics in clinical practice. Mackenzie’s three dimensions are elaborated below:

*Self-determination* involves possessing external freedom and opportunities to make choices and enact decisions. This dimension identifies external conditions for autonomy, namely freedom conditions (the social and political constraints that may interfere with the exercise of self-determination) and opportunity conditions (the necessary social environment that allows one to have choices).

*Self-governance* indicates the possession of internal capacities necessary to make choices and enact decisions. This dimension includes internal conditions of autonomy, namely competence conditions (such as cognitive capacity, responsiveness, etc.,) and authenticity conditions (the personal identification of one’s will, nonalienation from the social context, etc.).

*Self-authorization* refers to oneself as having the normative authority to exercise practical control over one’s life. This dimension incorporates conditions of autonomy related to accountability, self-evaluative attitudes, and social recognition.

In sum, Mackenzie’s proposal is a multidimensional and context-sensitive conceptualization of autonomy. She goes far beyond rational conditions as being sufficient for autonomy, instead incorporating other factors related to social embeddedness. Nevertheless, it must be noted that this typology hardly mentions the role of emotions and embodiment, important factors that Mackenzie does develop in other works [57, 58]. Feminist authors stress that a relational anthropology, sensitive to interdependency and vulnerability, pays more attention to how bodily and emotional factors influence decision making [59]. They claim that these additional aspects do not undermine a patient’s capacity for autonomous actions; on the contrary, they bring to the fore their role in coming to a more realistic understanding of autonomy [30, 31].. Anita Superson, for instance, reflects on Mackenzie’s idea of how the body is not something we simply own and use (“bodily ownership”); but something that shapes and is shaped by the choices that we can actually make in a particular situation (“bodily perspective”) [60]. Christine Tappolet, for her part, analyses the epistemic value of emotions in moral judgments since, she explains, emotions have cognitive content and therefore can conflict with conceptually articulated states, such as beliefs and judgments [61]. Our illustrative case confirms that not only relationships, but also emotions and bodily mediated experiences, are essential elements of decision-making processes that go beyond rational factors.

*In our case, Mr. Philip’s vacillating wishes are acknowledged and are understood to be part of his moral experience and his process of negotiating personal identity. Caregivers’ primary role, then, is not to decide which of these multi-faceted wishes is right from a rational point of view. Rather, it is to understand how these personal preferences interact and influence each other. Seemingly contradictory statements reveal tensions in a patient’s moral experience and therefore require respect and support rather than judgement and rejection.*

*Additionally, the three sets of conditions presented by Mackenzie complement the picture of autonomy, specifically the social and contextual factors. First, the conditions related to* self-determination *demand that Mr. Philip has feasible alternatives at hand, and that he exists within the socio-political framework to carry them out. Second, among the conditions for* self-governance*, Mr. Philip has to show rational capacities to understand, use, and evaluate factual information. Furthermore, he should be able to relate to others how his decision articulates with his own principles, values, and goals. Third, conditions about* self-authorization *require that Mr. Philip has a minimal threshold sense of self-trust and social recognition. The latter set of conditions aims to ensure that certain independence from social expectations is met. One step further, the healthcare team should explore the extent to which the lived reality of embodiment (revealed by the burdensome impact of his rectal bleeding) and of emotions (exemplified in the acrimonious discussion with his sister) plays a role in Mr. Philip’s autonomous capacity.*

2. ***Importance of relationships in autonomy***

The second shortcoming we presented above highlighted that relationships greatly impact autonomy, and that this factor has been commonly neglected in mainstream conceptualizations of autonomy. Today, in the wake of feminist and communitarian criticisms, it is no longer acceptable to think about autonomy in hypothetical, isolated situations. Currently, most theories take into account the social conditions of autonomy and are somehow relational, at least in a broad sense [62]. The critical point is how social embeddedness is conceptualized [17]. Thus, efforts should not be primarily directed toward “liberating” patients from their social environment. Rather, attention should be focused on how people relate to each other and how these relationships enhance or impede the proper exercise of autonomy. What are the primary relationships at stake in end-of-life situations?

**Fig. 1:**
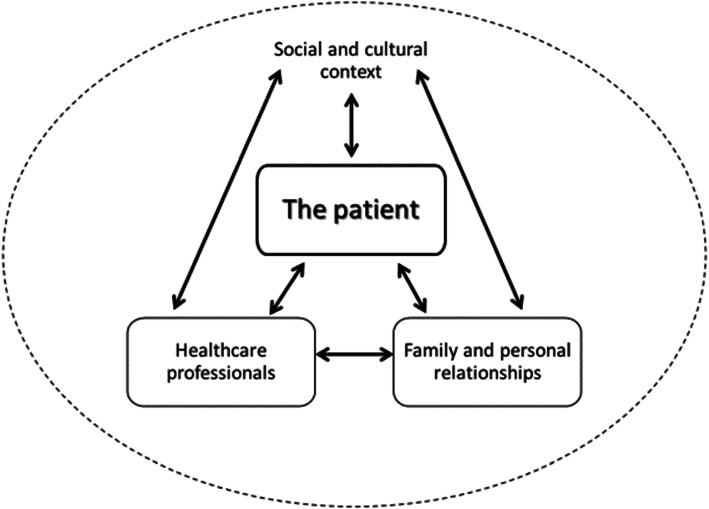
Schematic diagram of a relational approach to patient autonomy in end-of-life decision-making. Contextualized understanding of relational autonomy emphasizes interactions of primary stakeholders and the influence of sociocultural context

When analyzing end-of-life practices, four main stakeholders can be identified: The *patient* is situated in the center, directly interacting with the *healthcare team* and the *relational environment* (i.e. family, friends, communities). Moreover, there is a significant interplay between healthcare professionals and the relational environment, which will eventually affect the patient. These interactions do not occur in a void but rather in a particular *sociocultural context* that shapes them. Relationships, along with expectations and constraints, are all conditioned by the social and cultural framework. A relational account of autonomy addresses the complexity of these multiple interactions and acknowledges that they are embedded in a sociocultural context (Fig. 1).

#### Healthcare professionals

A relational approach to autonomy requires that the relationship between healthcare professionals and patients be redefined [63, 64]. Relational theorists argue that the current individualistic model of autonomy is unduly shaped by a contractual viewpoint and a consumer approach [65, 66]. They claim that the pendulum has swung from a paternalistic view to an overly liberal one [65]. Alternative forms of this relationship would be better based on friendship [66] and covenant [34, 65].

From a relational perspective, Stoljar advocates a new role for healthcare providers, one more committed to promoting patients’ autonomy rather than simply securing informed consent [67]. This new role includes paying special attention to social factors and creating special conditions in which patients can be more autonomous. In the same vein, relational theorists claim that mainstream interpretations of autonomy have overly emphasized the *right to non-interference* regarding those being cared for, essentially dismissing the *duty of responsibility* of the carers [68, 69]. In highlighting a practical outcome of this imbalance, Walter and Ross suggest that hyper-respecting patients’ autonomy is sometimes used by doctors as an “excuse” to avoid difficult situations when the patient or their family asks them directly for guidance [31]. In a relational model of care, by contrast, doctors are trained to offer recommendations without undermining the patient’s autonomy [64, 70].

Nurses also have a particular role to play in this regard, one consistent with the ideals of enhancing the well-being of patients and their families [22]. Their position in the sphere of clinical care encourages them to cultivate specific ethical knowledge, bearing a relational and embodied perspective [71]. The closeness of nurses vis-à-vis patients and families places them in a privileged position to play the role of assessors, information providers, supporters, and educators [22].

#### Family and other personal relationships

Mainstream discussions about autonomy tend to consider the interest of patients in isolation from their social context. However, real-world end-of-life situations do not occur in a purely individualistic closed-off setting but rather occur in an interpersonal setting. These situations have disruptive effects on the next of kin, especially on those who are expected to provide care [66]. Given our relational identity and intertwined interests, decisions that truly enhance the autonomy of all the participants are made in a way that promote responsibilities toward others who are affected by these decisions [72].

Relational theorists react against the current ethical and legal framework generally conceived of as a dyad between patient and physician; however, a patient-doctor-family triad seems more appropriate in characterizing what actually happens in clinical practice [40]. In the triad model, the family is not necessarily perceived of as a threat to autonomy. On the contrary, as Blustein concludes: “… family members, by virtue of their closeness to and intimate knowledge of the patient, are often uniquely well qualified to shore up the patient’s vulnerable autonomy and assist him or her in the exercise of autonomous decision-making” [73].

Also, a patient’s interpersonal relationships do not end at the boundary of the family. Other particular relations such as friends and religious or secular communities also deserve special attention. The possibilities of positive or negative interplay are endless. Real-life complexity requires a specific assessment of each case and the flexibility to respond to diverse situations [74]. Nevertheless, from a relational point of view, the default assumption should not be a suspicious glance toward personal relationships, as if patients accepting others’ interests were necessarily under undue pressure [72].

#### Social and cultural context

Relational authors highlight that the construction of personal identity has relational dimensions [29, 62]. This recognition has led to the conviction that social and cultural context play an important role in decision-making processes, facilitating or impeding the capacities that persons need for autonomous agency. Especially in end-of-life situations, greater awareness of the socio-political aspects is necessary, a step that moves ethical reflection beyond the private sphere of the clinical encounter between the doctor, the patient, and the family [75].

From another perspective, some authors argue that the excessive emphasis placed on the individual may have diminished due attention on social responsibility. In this vein, Meulenberg and Schotsmans criticize the overuse of self-determination, a stance that pretends to free the person from social bindings. This position may contribute to unwanted social consequences, such as the growth of social indifference and individual narcissism. Based on Hannah Arendt’s political philosophy, the authors maintain that the privatization of value judgments eventually leads to a diminution of engagement in and influence of the public sphere [76].

Lastly, relational theories are particularly sensitive to the way in which culture shapes care relationships. Especially, non-Western authors argue that complementary reflection on alternative perspectives is required, such as family-determination (vs. self-determination) and harmonious dependence (vs. independence) [77]. Those contexts deserve further attention in which the family is considered to be the decision-making unit [23, 78]. This approach will have consequences for clinical practices, such as truth-telling, informed consent, and advance-care planning. In this regard, Chan defends the idea that developing a form of moderate *familialism* may reduce conflicts between the patient, healthcare providers, and the family; thus, promoting peaceful co-existence in pluralistic societies [79].

#### Patient’s self-reflection and personal identity

‘Patient-centered care’ is a major emphasis of modern healthcare, and it has been suggested that relational autonomy is an essential component of it [80]. This kind of care aims to be “respectful of and responsive to individual patient preferences, needs, and values” ensuring “that patient values guide all clinical decisions” [81]. For this to occur, however, patients need to engage in a certain degree of self-reflection in order to identify their own needs and values. Only then, will autonomous actions be expressions that are consistent with one’s self-identity and individuality [49, 67].

However, relational theorists maintain that humans are not ideal self-transparent beings, ones with immediate knowledge of their own preferences and values [56, 76]. They stress the fundamental inaccessibility of the human self and maintain that one’s identity is not something discovered alone, nor does it occur only once forever [29, 50]. Self-discovery is not about listening to an “inner voice,” which reveals an essential, unchanging self; rather, it is an ongoing process of self-creation in constant interactions with others.

In sum, patients facing end-of-life situations are embedded in a dynamic process of self-creating their personal identity, which occurs over time. In this process, they find themselves at the center of relationships with healthcare professionals and their own personal environment. All these interactions are shaped by a particular sociocultural context that, in turn, shapes the patient’s exercise of autonomy. A relational view of autonomy takes into account these interactions and their respective impact, especially at the end of life when patients are more vulnerable and where these external factors may have greater influence.

*The importance of personal relationships in Mr. Philip’s exercise of autonomy becomes evident when the doctor, priest, and his sister play out their influential roles in decision-making regarding Mr. Philip’s wishes. We avoid a reductionist view of this influence as if they inevitably were cases of undue pressure. A relational understanding of autonomy reveals that Mr. Philip is in a process of self-discovery, a necessary activity in making autonomous decisions consistent with his personal values. In that exercise, other people have a legitimate role. First, the doctor is not supposed to exclusively provide neutral information. On the contrary, she is asked to exercise a guiding role using her medical expertise. In this guidance, she should also bring patient-specific knowledge into the relationship that was created through her personal interactions with Mr. Philip and his social environment. We also need to consider the contributions of nurses, psychologists, and other healthcare professionals who could offer complementary information. The priest and sister also play their proper roles as advisors, as long as Mr. Philip initiates the request for their advice, and as long as he maintains sufficient independence such that he can temper or reject their advice.*

*The default position should be to consider these relations as positive forces, not threats to the patient’s autonomy. From a relational perspective, then, these personal interactions may enhance Mr. Philip’s autonomy, as long as they help him to “discover” his desires, ones that align with his personal values. In this regard, the primary focus should be on properly articulating each one’s role as a doctor, priest, or relative, not on “freeing” him from his social environment.*

*Finally, a reflection may emerge if one detail is added to the case. What if Mr. Philip were an African immigrant residing in Belgium, rather than a native-born citizen? In this case, many ethicists would probably be more willing to give weight to the family’s wishes and/or their religious beliefs. Nevertheless, it should be remembered that cultural context has a weighty role to play in decision-making in a relational perspective of autonomy.*

3. ***A scalar conceptualization of autonomy***

The third criticism of an individualistic view of autonomy addressed the problem of characterizing autonomy in a binary way (one having either complete autonomy or no autonomy). Relational theories of autonomy, on the other hand, tend to conceptualize autonomy as represented in a continuum, with scalar properties (one having a degree of autonomy) [29, 48, 51]. This option captures better the fluctuating nature of autonomy in end-of-life care and it is therefore context-sensitive [22, 25]..

In the case of patients at the end of life, even though their autonomy may be compromised to some degree, it still deserves respect and consideration [49, 82]. Physical and cognitive fragility can diminish decision-making and executive function. However, when these capacities are compromised, one’s autonomy can be exercised with the assistance of others [25]. From a relational perspective, healthcare professionals can strengthen patient autonomy by compensating for diminutions related to illness and dependency, thereby assisting them in preserving their sense of self and dignity [10, 83]. In this regard, relational theorists insist that respecting patients’ autonomy should not diminish doctors’ responsibility toward patients [34, 62]. Thus, a scalar notion of autonomy, more context-sensitive, implies that doctors’ responsibility to provide the best treatment takes different forms depending on whether the patient is completely autonomous or is autonomous to a lesser degree. Along these lines, Killackey and colleagues maintain that “a relational view of autonomy allows a broader account of personhood that recognizes the subtleties of autonomy and includes a range of capacities, which can still be expressed within and through relationships of vulnerability and dependence” [21].

Some authors argue that vulnerability is present not only with patients but also with healthcare professionals [22]. In this sense, Boldt defines vulnerability in a way that symmetrizes the healthcare relationship between healthcare provider and patient [84]. Healthcare providers are affected by a “cognitive vulnerability” from the moment they are required to engage in a process where basic values and personal identities are at stake. A rejection of this shared vulnerability may explain why some doctors avoid giving their opinion, sometimes protecting themselves under the guise of respect for patient autonomy [31].

In sum, we consider autonomy to be a scalar concept, rooted in a non-idealized person with interrelated capacities and vulnerabilities. This perspective considers patients to be more or less autonomous, not possessing complete autonomy or lacking autonomy.

*In the case of Mr. Philip, considering the scalar character of autonomy may be useful in two ways. First, a terminal systemic weakness diminishes his capacities in a significant way. Thus, it is difficult to view him as a completely autonomous patient. However, although his autonomy may be partial and compromised, it is not absent. His actual autonomy should be respected as well. In this sense, Mr. Philip’s vulnerable state lays the foundation for a duty of responsibility and care on the part of the patient’s environment, which includes the healthcare team, the priest, and his sister. Second, a shared and symmetrical understanding of vulnerability emphasizes the need to care for the caregiver as well. The healthcare team is also involved in a process of personal discovery, through which Mr. Philip’s authentic wishes are achieved through dialogue of stakeholders. In this process of common search, they share uncertainties and deserve particular care.*

4. ***Temporally extended and process-oriented approach to autonomy***

The fourth shortcoming considers autonomy to be a dynamic process “in motion”, not a static capacity of discrete actions [50]. Although a narrow conceptualization of autonomy focused on isolated decisions may align well with practical implementations, such as informed consent, the exercise of autonomy in real-world situations is better characterized as an evolving process that changes depending on the circumstances [85].

In this regard, some authors prefer to consider autonomy from a temporal perspective unfolding over time [17, 22]. They pay special attention to the ongoing and interactive process of reconstructing autonomy through a constant interplay with others [48, 85]. Autonomy in end-of-life situations may be compromised, yet it still can be promoted by healthcare providers as long as there has been time and continuity to become acquainted with the patient’s values and goals [83]. Regular encounters with patients and families can enrich the staff’s knowledge about patients’ preferences and eventually will help them interpret their wishes when it becomes difficult for patients to express their desires.

Finally, Baumann notes another advantage of a diachronic vision of autonomy [17]. Considering autonomy to be a process evolving over time may softens a static vision of autonomy in which patients are immutably “trapped” in their own decisions. Adopting a diachronic view, on the other hand, allows the patient to consider the possibility of “autonomous change” and of “autonomous emancipation” [17]. The former means that a decision can always be reconsidered, something that is relatively common in end-of-life situations, and still be considered an autonomous choice. The latter includes the possibility for the patient to resist social and group expectations that may be imposed against their authentic desires. Thus, a diachronic perspective of autonomy better integrates criteria of consistency and durability over time.

*Applied to our case, Mr. Philip’s exercise of autonomy is better understood as a process occurring over time. It would be arbitrary to stop the “timer” and consider his final decision to be the one voiced after his conversation with the medical doctor, the priest, or his sister. These three moments comprise the process of autonomy-building when making a complex decision. From a diachronic point of view, it is clear that Mr. Philip’s decision is still not stable or consistent; therefore, the healthcare team should allow more time to pass until the decision becomes stable. One may reasonably question whether it is possible to obtain certainty about when the decision is considered binding. It all depends on the seriousness of the decision and the urgency of the medical condition. In our case, because euthanasia is an irreversible act, prudence demands that the healthcare team wait and help Mr. Philip to make up his mind. From a diachronic perspective of autonomy, the decision should eventually be revisited again after significant conversations.*

### Procedural application to the case

Our 2019 systematic review revealed that a significant distance exists between theoretical accounts of relational autonomy and their practical implementation [24]. In order to explain this distance, several authors suggest that an individualistic account of autonomy is generally related to a negative concept of freedom (i.e. *freedom from*); while relational autonomy aligns more often with a positive concept (i.e. *freedom to*) [51, 67, 68]. The focus on negative obligations lends itself better to practical implementation (e.g., informed consent) and legal standards (e.g., patient rights). On the other hand, a focus on positive obligations, one promoting relations that are meaningful for individuals in particular contexts, are more difficult to translate into general rules or clearly articulated procedures [47, 82, 86].

Aware of this constraint, we now propose a procedural application of relational autonomy to Mr. Philip’s end-of-life situation and request. Inspired by Tonelli and Misak’s model in cases of compromised autonomy [25], we also aim at responding to the four shortcomings revealed by our analysis of lived experiences of autonomy. Our concrete proposal consists of three steps:

*First, the medical staff drafts a course of action for Mr. Philip based on his best interests. The complementary views of nurses, the psychology support team, and the pastoral care service are systematically integrated. This frame-document brings into explicit dialogue the clinical knowledge of a multidisciplinary team and the patient’s goals of care. These goals have been learned through personal contact with Mr. Philip and his relational environment over time*.

*Second, Mr. Philip makes end-of-life care decisions. If these decisions fit within the boundaries of the frame-document, they can be honored. If they do not, the patient’s autonomy needs to be reassessed. The role of Mr. Philip’s relational environment and his internal capacity for decision-making, including cognitive, emotional, social, and embodied factors, are all re-evaluated. While doing so, the healthcare team revisits Mr. Philip’s long-standing preferences and goals. This is a process of authentication on how the decision aligns with his underlying values. Responsiveness and accountability are key elements to consider in order to ensure alignment with the patient’s personal life and moral beliefs. Likewise, a reasonable amount of time is allowed to pass in order to thoroughly analyze the consistency and stability of the decision.*

*Third, the decision’s moral weight is reassessed. If Mr. Philip meets the previous requirements in a global way, his decision can be acknowledged as authentic and stable, even if it does not agree with the best interests assessment arrived at by the clinicians. However, if Mr. Philip does not satisfactorily meet these criteria, his decision is not deemed to be compelling. The euthanasia act should be suspended, and further discussions between the healthcare team, the patient, and the relational environment are begun.*

This proposal can be seen as a prudent approach that reconciles the principle of autonomy with other moral and professional principles (e.g., care, relationality, responsibility) through a process of engagement and negotiation [87]. Guided by a “logic of care,” rather than blindly adhering to a “logic of choice,” it thus legitimates some interference with personal choices under certain control [88]. By doing so, it seeks neither to abandon people to the hazards of their own choices under the guise of autonomy, nor alternatively to apply restrictive practices in an arbitrary manner [87].

Some may object that our proposal is a complex and time-consuming procedure. Nevertheless, a similar procedure is commonly followed, although implicitly [25]. Producing a formal written assessment seeks to increase the process’ transparency, although it might be necessary only for relevant end-of-life decisions (i.e. morally weighty decisions). With ordinary treatment decisions, this tool may serve as background guidance that helps to promote a relational and more complex account of autonomy in end-of-life practices.

## Conclusions

In this article, we put into direct dialogue two often-unrelated medico-ethical matters: lived experience of different stakeholders at the end of life and conceptual theories of relational autonomy. First, we uncovered and analyzed four shortcomings of the mainstream interpretation of autonomy when applied to lived reality in end-of-life practices. Second, we developed a positive concept of relational autonomy that responds to these four shortcomings, drawing on different ethical approaches in doing so. Building on the outcomes of these two efforts, we proposed a specific procedure to implement relational autonomy in end-of-life practices. Our proposal incorporates multidimensional, socially embedded, scalar, and temporally extended aspects of relational theories of autonomy. Finally, the analysis of a case that served as a touchstone throughout the article, has shown how a relational account of autonomy can be implemented in real situations at the end of a patient’s life.

We conclude with four considerations about relational autonomy and thus make explicit some principles that have guided our reflection in this article.

### First, a bottom-up approach

We have thought about autonomy inductively, starting from a consideration of real-world lived experiences. The relational turn aligns better with what Jennings calls an *ideographic* approach (from practice to theory; from lived reality to interpretations of right and wrong) rather than a *nomothetic* approach (from theory to practice; from normative theories to practices of decision-making in medicine) [50].

### Second, an option for complexity

We acknowledge that the issues laid out and solutions offered in this paper make autonomy more complex and less neat. We believe this is a fair price to pay when focusing on “actual autonomy” and not on “ideal autonomy” [49]. Agich acknowledges that “focusing on actual autonomy brings ethical reflection to bear on the mundane, interstitial, ongoing reality, rather than on idealized crises or problems” [49]. Rather than creating neat procedural outcomes for hypothetical ethical conflicts, relational ethics is particularly helpful in guiding day-to-day ethical situations that occur between real people [86].

### Third, the search for a strong anthropology

We are convinced that a proper accounting of relational autonomy demands a strong anthropology: i.e. a more integral view of the person, one capable of providing normativity to the ethics derived from it. Narrow accounts of autonomy conceive it as an exercise of self-determination made by self-transparent agents in ideal circumstances. Nevertheless, “autonomy, as lived, is a relational experience that involves both independence from others and dependence on others” [89]. A strong relational anthropology acknowledges that human beings are not self-transparent agents. On the contrary, there is always a fundamental inaccessibility of the human self [29]. Thus, patients’ indecisiveness and ambivalence express “movements” within the core of the person, and it is especially there where *the other* plays a role in the process of self-discovery.

### Fourth, we move away from individualism, not from the value of individuality

This distinction has been repeatedly expressed by relational theorists [22, 29, 50]. The principle of ‘respect for autonomy’ is a paramount achievement of modernity and should not be rejected wholesale. Rather, it is the individualistic interpretation of this principle that is being criticized by these authors. Thus, they suggest that it is necessary to make a “shift in emphasis” when characterizing autonomy, which draws attention to other morally significant considerations previously neglected [90]. One may question, then, how far this turn of relational autonomy should go. Whether a paradigm shift or just a refocus, a relational account of autonomy is an approach that can be applied successfully not only to end-of-life care practices, but also to other settings like pediatrics or dementia care, where decision-makers are not fully competent persons [91, 92].

## Data Availability

Not applicable.

## Footnotes

1 Talking about a ‘mainstream interpretation of autonomy’, a ‘traditional view’, or a ‘default position’ is a delicate issue, since there is no single canonical interpretation of this principle. Nevertheless, many authors refer to these or similar notions, often to criticize them. Generally speaking, Beauchamp and Childress’s work is taken as the chief reference. It should be noted that their position has evolved throughout their long academic careers. In this paper, we distinguish when necessary the authors’ personal position from others’ interpretation.

2 This illustrative case portrays a fictional character. The case is inspired by the clinical experience of the first author (CGV) in end-of-life contexts. It is therefore real-life based but does not correspond to any particular person.

3 In the context of this article, we use ‘rational’ in a narrow sense – in line with the default autonomy view. As such, rationality, referring to a form of argumentation that is based upon evidence and logical reasons, stands over against emotional or intuitive ways of arguing. The underlying rationalist view neglects that human beings cannot be reduced to their mind, but consist of heart and body as well.
